# Correlation between the Potency of Flavonoids on Mushroom Tyrosinase Inhibitory Activity and Melanin Synthesis in Melanocytes

**DOI:** 10.3390/molecules23061403

**Published:** 2018-06-09

**Authors:** Worrawat Promden, Wittawat Viriyabancha, Orawan Monthakantirat, Kaoru Umehara, Hiroshi Noguchi, Wanchai De-Eknamkul

**Affiliations:** 1 Division of General Science, Faculty of Education, Buriram Rajabhat University, Buriram 31000, Thailand; 2 Bureau of Drug Control, Food and Drug Administration, Ministry of Public Health, Nonthaburi 11000, Thailand; wittawat.vi@gmail.com; 3 Division of Pharmaceutical Chemistry, Faculty of Pharmaceutical Sciences, Khon Kaen University, Khon Kaen 40002, Thailand; oramon@kku.ac.th; 4 School of Pharmaceutical Sciences, University of Shizuoka, Shizuoka 422-8526, Japan; kaoru.umehara@hamayaku.ac.jp (K.U.); h-noguchi@nichiyaku.ac.jp (H.N.); 5 Faculty of Pharmaceutical Sciences, Yokohama University of Pharmacy, Yokohama, Kanagawa 245-0066, Japan; 6 Department of Pharmacognosy, Nihon Pharmaceutical University, Kitaadachi, Saitama 362-0806, Japan; 7 Natural Product Biotechnology Research Unit, Department of Pharmacognosy, Faculty of Pharmaceutical Sciences, Chulalongkorn University, Bangkok 10330, Thailand

**Keywords:** tyrosinase, flavonoid, *Dalbergia parviflora*, melanogenesis

## Abstract

Twenty-seven flavonoids isolated from *Dalbergia parviflora* with vast structural diversity were screened for inhibitory activity against mushroom and murine tyrosinases using l-DOPA as the substrate. Among the flavonoids tested, only four—khrinone (**5**), cajanin (**9**), (3*RS*)-3′-hydroxy-8-methoxy vestitol (**21**), and (6a*R*,11a*R*)-3,8-dihydroxy-9-methoxy pterocarpan (**27**)—reacted with mushroom tyrosinase, with IC_50_ values of 54.0, 67.9, 67.8, and 16.7 μM, respectively, and only compound **27** showed inhibitory activity against murine tyrosinase. With cell-based assays, only compounds **9** and **27** effectively inhibited melanogenesis in B16-F10 melanoma cells (by 34% and 59%, respectively), at a concentration of 15 μM, without being significantly toxic to the cells. However, the crude extract of *D. parviflora* and some of the flavonoid constituents appeared to increase melanin production in B16-F10 cells, suggesting that there are flavonoids with both inhibitory and stimulatory melanogenesis in the crude extract. Studies on the correlation between the enzyme-based and cell-based assays showed that only the flavonoids with IC_50_ values below 50 μM against mushroom tyrosinase could inhibit the mammalian tyrosinase, and thus, reduce melanogenesis in B16-F10. Flavonoids with the IC_50_ values greater than 50 μM, on the other hand, could not inhibit the mammalian tyrosinase, and had either no effect or enhancement of melanogenesis. In conclusion, the tyrosinase enzyme from mushroom is not as selective as the one from mammalian source for the enzyme-based melanogenesis inhibitory screening, and the mammalian cell-based assay appears to be a more reliable model for screening than the enzyme-based one.

## 1. Introduction

Tyrosinase (EC 1.14.18.1) is the key enzyme involved in melanin biosynthesis in living organisms. In animal cells, tyrosinase oxidizes l-tyrosine to 3,4-dihydroxyphenylalanine (l-DOPA), and subsequently, l-DOPA to DOPA quinone, in the initial steps in melanogenesis, a process which is mainly responsible for dark skin color. In plant-derived foods, tyrosinase catalyzes the oxidation of phenolic compounds to their corresponding quinones, and is responsible for the enzymatic browning of fruits and vegetables [1]. Therefore, tyrosinase inhibitors have become increasingly important in the cosmetic field as skin-whitening agents and in the food industry [2,3,4,5].

Mushroom tyrosinase has been widely used as a substitute for mammalian tyrosinase to screen for tyrosinase inhibitors as it is inexpensive and commercially available in a purified form [5]. Several plant flavonoids have been reported to have varying degrees of inhibitory activity towards mushroom tyrosinase, and are thought to be potential inhibitors of melanin synthesis in mammalian melanocytes [6,7,8]. However, there is a limitation of information about the correlation between the inhibitory activity of mushroom tyrosinase by flavonoids and the actual inhibitory effect of melanin formation in mammalian cells. Only one report has indicated that the inhibition rate of mushroom tyrosinase might not accurately reflect the inhibition rate of melanin synthesis in melanocytes [9]. We have reported, previously, the isolation and structure of estrogenic-like compounds from the heartwood of *Dalbergia parviflora*, a Thai folk medicine traditionally used as a blood tonic and menstruation normalizer. More than 60 flavonoids were isolated and identified, including 22 isoflavones, 12 isoflavanones, 10 isoflavans, six flavonones, four flavanonols, and six pterocarpans [10,11]. The structure–antioxidant relationship in the isoflavonoids from *D. parviflora* has also been reported [12]. The structural diversity of the flavonoids has provided a good source for studying the correlation between enzymatic assay and cell-based assays.

In this study, crude extract and 27 flavonoids prepared and isolated from the heartwood of *D. parviflora* were tested for their ability to inhibit mushroom and murine tyrosinases using l-DOPA as the substrate. The inhibition of mushroom tyrosinase was then compared to the inhibition of crude murine tyrosinase extracted from B16-F10 melanoma cells. Finally, the effects of these compounds on the overall melanin production in B16-F10 melanoma cells were studied to observe the correlation between the results of the enzyme-based and cell-based assays.

## 2. Results and Discussion

### 2.1. Inhibitory Effect of Flavonoids on the o-Diphenolase Activity of Mushroom Tyrosinase

The structures of the 27 flavonoids isolated from *D. parviflora*, grouped into isoflavones, flavanones, isoflavanones, isoflavans, and pterocarpan, are shown in Table 1. Each compound (200 μM) was screened for *o*-diphenolase inhibitory activity of mushroom tyrosinase using l-DOPA as the substrate. A total of 11 active inhibitors from the preliminary screening were then determined for their 50% of inhibition (IC_50_) values, and the results are summarized in Table 2.

From the nine isoflavones (compounds **1**–**9**), only two compounds with a hydroxyl group at the C-2′ position of ring B—khrinone B (**5**) and cajanin (**9**)—exhibited *o*-diphenolase inhibitory potential, with IC_50_ values of 54.0 ± 6.0 and 67.9 ± 6.2 μM, respectively. The other five isoflavones—namely formononetin (**1**), calycosin (**2**), biochanin A (**3**) 3′-O-methylorobol (**6**), and khrinone C (**7**)—did not show inhibitory activity on mushroom tyrosinase. In contrast to a previous report, calycosin has been shown to be a monophenolase inhibitor of mushroom tyrosinase with an IC_50_ value of 38.4 μM. In addition, calycosin reduced melanin biosynthesis in murine melan-a melanocytes [13]. Most of the isoflavanones, isoflavans, and flavanones showed poor or no inhibitory activity on tyrosinase. (3*R,S*)-3′-hydroxy-8-methyoxy vestitol (**21**), an isoflavan with hydroxyl groups at the R2′and R3′ positions of ring B, showed inhibition with an IC_50_ value of 67.8 ± 5.8 μM. Pterocarpan (**27**) was the best mushroom tyrosinase inhibitor among the 27 flavonoids tested, with an IC_50_ value equal to kojic acid (16.7 ± 5.0 μM and 16.8 ± 4.8 μM, respectively). Moreover, the positive control, oxyresveratol, exhibited the strongest inhibitory effect on mushroom tyrosinase with an IC_50_ of 0.19 ± 0.06 μM. The crude extract of *D. parviflora* exhibited tyrosinase inhibitory activity with an IC_50_ of 2.6 ± 0.4 μg/mL.

The inhibition of enzymes by certain flavonoids may be due to the interaction of the flavonoids with free radicals generated at the active sites of the enzymes [14] or with copper ion in the catalytic domains of the enzymes [7]. The numbers and positions of the hydroxyl groups attached to the flavonoids skeletons are the key to the inhibitory activity of mushroom tyrosinase. Chang et al. found that 6,7,4′-trihydroxyisoflavone, glycitein (6-methoxy-7,4′-dihydroxyisoflavone), daidzein (7,4′-dihydroxyisoflavone), and genistein (5,7,4′-trihydroxyisoflavone) inhibited the monophenolase activity of mushroom tyrosinase with IC_50_ values of 9.2, 237, 264, and 822 μM, respectively. The results suggest that the hydroxyl groups at the C6 and C7 positions of ring A of the isoflavone skeleton might play an important role in the expression of monophenolase inhibitory activity [15]. Chang et al. also found that 7,8,4′-trihydroxyisoflavone and 5,7,8,4′-tetrahydroxyisoflavone irreversibly inhibit the monophenolase and diphenolase activities of tyrosinase. The study suggested that the presence of hydroxyl groups at the C7 and C8 positions of the isoflavone skeleton may also cause this irreversible inhibition [16]. 

In the series of flavonoids in this study, only (3*S*)-8-demethylduartin (**23**) contained 7,8-dihydroxy groups. However, the inhibitory activities of khrinone B (**5**) and cajanin (**9**) suggested that not only the 6,7- or 7,8-dihydroxy groups of ring A, but also the hydroxyl group at the C2′ position of ring B of isoflavone or isoflavanone skeletons, might play important roles in the expression of *o*-diphenolase inhibitory activity. The inhibitory activities of (3*RS*)-3′-hydroxy-8-methoxy vestitol (**21**) and (6a*R*,11a*R*)-3,8-dihydroxy-9-methoxy pterocarpan (**27**) supported the importance of both the hydroxyl group at the C3′ position on ring B of isoflavans and pterocarpan for *o*-diphenolase inhibitory activity.

In this study, the 7,8-dihydroxy groups are only present in (3*S*)-8-demethylduartin (**23**), which could be a potent tyrosinase inhibitor. However, after pre-incubation of (3*S*)-8-demethylduartin (**23**) with the enzyme in the absence of the substrate (l-DOPA), an orange metabolite with an optical density at 475 nm was observed. This observation suggests that (3*S*)-8-demethylduartin (**23**) can act as a chromogenic substrate analog for mushroom tyrosinase. The other flavonoids that were found to be chromogenic substrate analogs include genistein (**4**), tectorigenin (**8**), and (2*S*)-naringenin (**25**). These results are comparable to a previous report in which resveratrol, a phenylpropanoid, was oxidized by mushroom tyrosinase and converted to a newly oxidized compound which was observed at 475 nm and became a tyrosinase inhibitor [17,18]. Moreover, Gasowska-Bajger and Wojtasek demonstrated that the oxidation of quercetin, kaempferol, morin, catechin, and naringenin by mushroom tyrosinase strongly influenced the measurement of the absorbance at 475 nm during oxidation of l-tyrosine or l-DOPA, generating false inhibition or activation effects [19].

### 2.2. Inhibitory Effect of Flavonoids on the o-Diphenolase Activity of Murine Tyrosinase

The inhibition of murine tyrosinase was studied using the flavonoids at a concentration of 200 μM. The results revealed that among the 27 flavonoids, only *(*6a*R*,11a*R*)-3,8-dihydroxy-9-methoxy pterocarpan (**27**) showed a weak inhibitory effect on the *o*-diphenolase activity of murine tyrosinase with an inhibition of 29.2 ± 2.9% (Table 3). The crude extract and the other flavonoids did not show any inhibition (data not shown), whereas 200 μM of kojic acid and oxyresveratrol, the positive controls, showed strong inhibitory effects of 73.8 ± 1.0% (IC_50_ = 39.9 μM) and 99.4 ± 0.9% (IC_50_ = 0.88 ± 0.16 μM), respectively.

Notably, the flavonoids exhibited stronger effects on mushroom tyrosinase than murine tyrosinase activity. In fact, almost all studies conducted on tyrosinase inhibition have used the commercially available champignon mushroom *(Agaricus bisporus)* tyrosinase. Although these results may highlight that mushroom tyrosinase can be used for anti-tyrosinase activity screening, it does not appear to be sufficiently accurate to measure the potential of the active substance as a whitening agent. A more appropriate method would be to use the mammalian tyrosinase inhibition assay and melanogenesis inhibition results from the cell-based assay. However, mushroom tyrosinase is still a suitable screening tool to measure the anti-browning activity of plant-derived foods in the food industry.

### 2.3. Effects of the Flavonoids on Cell Viability and Melanogenesis of B16-F10 Melanoma Cells

**Figure 1:**
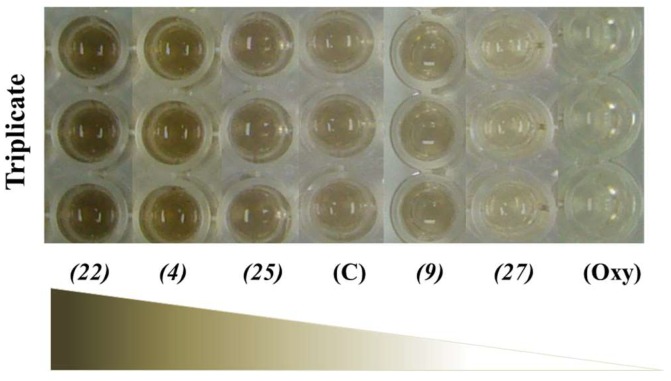
Total melanin content assay—the tested compound was added to a final concentration of 30 μM of B16-F10 melanoma grown cells. The compounds genistein (**4**), duartin (**22**), and (2*S*)-naringenin (**25**) increased the melanin content. The inhibitory effect of melanogenesis was found in cajanin (**9**) and (6a*R*,11a*R*)-3,8-dihydroxy-9-methoxy pterocarpan (**27**). Oxyresveratrol (Oxy) was used as a positive control. (C) is the negative control to which 0.5% dimethyl sulfoxide (DMSO) was added.

Murine B16-F10 melanoma cells were used as a model to examine the effects of the 27 flavonoids on cell viability and melanogenesis (Table 2). The melanin content was measured at 405 nm, a wavelength at which both pheomelanin and eumelanin absorb light [20]. The reason for using phenol red-free supplemented DMEM was that as cells grow, they produce lactic acid during cellular metabolism, which causes the pH of the medium to decrease. This decrease changes the phenol red DMEM medium to yellow, which interferes with the measurement of the brown melanin production recorded at 405 nm. The results of the dark-brown cell culture are shown in Figure 1, which led to the measurement of cell viability and total melanin content (Table 3).

Compounds **13**, **15**, and **26** exhibited cytotoxicity against melanoma cells at a concentration of 15 μM with the cell viability decreasing to approximately 70–80% of the control. Compounds **5**, **14**, and **21** showed toxicity at a concentration of 30 μM with a cell viability of 60%. The highest cytotoxicity was found in compound **27**, with a cell viability of 14% at 30 μM, but no toxicity was exhibited at 15 μM. Compounds **8**, **16**, **19**, and **22** increased the quantity of melanoma cells with a cell viability of 120–140% compared with the control at a concentration of 15 μM. The inhibitory effect of melanogenesis was found in compounds **9** and **27**. Although compound **27** exhibited 60% inhibition of melanogenesis at 15 μM without affecting the cell viability, it showed cytotoxicity at 30 μM (Table 3). Only compound **9** significantly decreased the cellular melanin content without affecting the cells at both concentrations.

**Figure 2:**
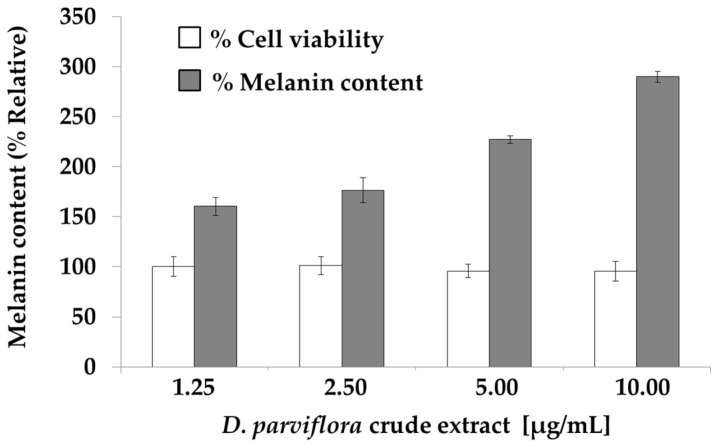
*D. parviflora* crude extract induced melanogenesis in B16-F10 melanoma cells. The cells were treated with the crude extract for 72 h. Data represent means ± SDs. The melanin content was significantly different between the control and treatment groups (*p* < 0.05).

From the 27 flavonoids tested, most of them increased melanin production, especially Duartin (**22**), which exhibited a three-fold increase in melanin content with respect to the control (Figure 1 and Table 3). Treatment of B16-F10 cells with the *D. parviflora* crude extract significantly promoted melanin formation in a dose-dependent manner. At the concentrations of 1.25, 2.5, 5.0, and 10.0 μg/mL of the crude extract, the melanin content increased by 158, 173, 226, and 288%, respectively, without exhibiting toxicity (Figure 2). However, at the concentrations of 20, 30, and 40 μg/mL, the cell viability decreased to 85, 80, and 75%, respectively.

A previous report indicated that quercetin and naringenin stimulate melanogenesis in cultured murine B16-F10 melanoma cells. Kubo et al. suggested that the improvement of melanin production by quercetin may be due, in part, to melanocytotoxicity [21], whereas Nagata et al. suggested that quercetin stimulates melanogenesis by increasing tyrosinase activity and decreasing other factors, such as melanogenic inhibitors [22]. Ohguchi et al. [23] and Bouzaiene et al. [24] also reported that naringenin increases the expression level of melanogenic enzymes in B16-F10. Takekoshi et al. reported that flavonoids, including quercetin, kaempferol, rhamnetin, fisetin, apigenin, luteolin, chrysin, and genestein, showed melanogenesis-promoting actions on human melanoma cells (MMV II), which promoted melanogenesis by flavonoids that was dependent on increased intracellular tyrosinase activity. Moreover, the relationship between the structure and melanogenesis-promoting actions of the flavonoids suggested that the hydroxyl group bound to the phenyl group plays an important role in the stimulation of melanogenesis [25]. In this study, the comparison of the chemical structures between duartin (**22**) and (3*S*)-8-demethylduartin (**23**) revealed that the 7,8-dihydroxy structure in the isoflavans was associated with melanogenesis inhibition. The R8-OMe substitution in duartin (**22**) had a strong effect on melanogenesis stimulation. The comparison of chemical structures between (3*RS*)-3′-hydroxy-8-methoxy vestitol (**21**) and duartin (**22**) also revealed that the 2′,3′-dihydroxy structure was associated with an inhibition of mushroom tyrosinase. Duartin (**22**), associated with 2′-methoxy structure, exhibited the strongest melanogenesis stimulation and loss of mushroom tyrosinase inhibitory activity.

All tyrosinases have a binuclear type three copper center within their active site in common. However, no common tyrosinase protein structure has been found to occur across all species [26,27,28,29]. There are significant differences between mushroom tyrosinase and mammalian tyrosinase in terms of structural properties, molecular properties, kinetic properties, and localization. Tyrosinase from the mushroom *A. bisporus* has been reported to be a soluble cytosolic enzyme containing two different subunits, referred to as heavy (H) and light (L), with molecular weights of 43 and 13.4 kDa, respectively. In aqueous solution, the predominant form of the mushroom tyrosinase is the quaternary L_2_H_2_ structure with a molecular weight of 120 kDa, whereas the active form appears to be L_2_H with an apparent molecular weight of 69 kDa [30]. In contrast, human tyrosinase is a monomeric membrane-bound glycoprotein with a molecular weight of 66.7 kDa [29]. Song et al. suggested that there is no significant correlation between the inhibition of mushroom tyrosinase with human melanoma tyrosinase and melanogenesis [31]. A study on the inhibition of mushroom tyrosinase and the cellular tyrosinase activities of mulberroside A, oxyresveratrol, resveratrol, and arbutin also suggested that the in vitro inhibition rate of mushroom tyrosinase might not represent the inhibition rate of melanin synthesis for in vivo systems [9]. However, we suggest that, in flavonoids, anti-tyrosinase activity against mushroom would only appear in parallel with mammalian tyrosinase activity and melanogenesis inhibition when the enzyme inhibition activity in mushroom tyrosinase is greater than 60% at a concentration of 200 μM or an IC_50_ lower than 50 μM. In other words, most flavonoids are likely to stimulate melanogenesis despite inhibiting mushroom tyrosinase activity.

## 3. Materials and Methods

### 3.1. Chemicals

3,4-Dihydroxy-l-phenylalanine (l-DOPA), kojic acid, oxyresveratrol, and mushroom tyrosinase were purchased from Sigma-Aldrich (St. Louis, MO, USA). The 27 flavonoids from *D. parviflora* were obtained from Kaoru Umehara [10,11].

### 3.2. Mushroom Tyrosinase Inhibitory Assay

The test samples, l-DOPA, and tyrosinase enzyme solutions were dissolved in 50% (*v*/*v*) dimethyl sulfoxide (DMSO) and 20 mM phosphate buffer solution (PBS) of pH 6.8, respectively. The *o*-diphenolase activity of mushroom tyrosinase was performed in 96-well plates using a modified method of that performed by Likhitwitayawuid and Sritularak [32]. The reaction mixture consisted of 140 μL of 20 mM phosphate buffer at pH 6.8, 20 μL of test sample solution, and 20 μL of mushroom tyrosinase (100 unit/mL, E.C. 1.14.18.1, Sigma). The mixture was pre-incubated at 25 °C for 10 min. Subsequently, 20 μL of 2.5 mM l-DOPA was added, and the mixture was incubated for 20 min at 25 °C. During the reaction, l-DOPA was converted to dopachrome, which resulted in a change in color from colorless to orange. This change was measured through absorbance at 475 nm. Instead of the sample solution, 50% DMSO was used as the control. The reaction mixture without enzyme served as a blank. The inhibitory activity of the sample was expressed as the concentration that inhibits 50% of enzyme activity (IC_50_).

### 3.3. Cell Cultures

*Mus musculus* (mouse) melanoma cell line, B16-F10 (ATCC^®^ Number: CRL-6475™, Manassas, VA, USA), was cultured in complete Dulbecco′s Modified Eagle′s Medium (DMEM, phenol red-free medium) containing 10% fetal bovine serum, 25 mM glucose, 4 mM l-glutamine, 1 mM sodium pyruvate, 10 μg/mL penicillin, and 10 μg/mL streptomycin, at 37 °C, with 5% carbon dioxide (CO_2_) in a humidified atmosphere.

### 3.4. Murine Tyrosinase Inhibitory Assay

B16-F10 melanoma cells were grown to 100% confluence on 10 cm dishes. The trypsinized cells were washed with cold phosphate buffer (20 mM, pH 6.8) and then disrupted in 1 mL of the buffer containing 1% Triton X-100. The cells were lysed by vortex and incubated at 4 °C for 1 h to solubilize the tyrosinase enzyme. The solution was centrifuged at 13,000× *g* for 15 min at 4 °C to remove the cell debris. The supernatant was dialyzed against 20 mM sodium phosphate buffer (pH 6.8) to remove the Triton X-100. The dialyzed solution was used as a source of crude murine tyrosinase enzyme. The solution contained 1 mg/mL protein, which was determined using a protein assay kit, based on the Bradford method (Bio-Rad Protein assay, Bio-Rad, Hercules, CA, US). The inhibitory activity was determined as described above for mushroom tyrosinase, but the incubation time was optimized at 2 h and 37 °C.

### 3.5. Cell Viability and Total Melanin Content Assay

Cell viability and melanin content assay were performed in quadruplicate in at least three independent experiments. Cell viability was taken as the reducing power of the living cells that reduce resazurin-based solution (PrestoBlue™, Invitrogen, Carlsbad, CA, USA) to resorufin, which results in a bright red color and is highly fluorescent. Briefly, B16-F10 cells were cultured in a 96-well plate at a concentration of 10,000 cells/0.1 mL/well. After incubating for 24 h, equal volumes of fresh medium containing various concentrations of the test samples were added to the cells and were incubated for 72 h. Cells were then washed with phosphate buffer, and then 0.1 mL of PrestoBlue™ solution in RMPI 1640 serum free media (1:10) was added to each well. After an hour, the fluorescence was quantified using spectrofluorophotometry (excitation 535 nm, emission 610 nm).

The total melanin content, produced both extracellularly and intracellularly, was determined as follows: B16-F10 cells were seeded onto a 24-well plate at a concentration of 50,000 cells/0.5 mL/well and were incubated for 24 h, followed by treatment with either the test compounds or the control (0.5% DMSO), and then incubated for 72 h. For extraction, the total produced melanin and 0.5 mL of 2 N NaOH containing 20% DMSO were added directly to the cell culture medium, mixed by pipetting and then incubated at room temperature for 1 h. The lysed cell extract was centrifuged at 13,000× *g* for 5 min. Finally, the supernatant was transferred to a 96-well plate and the relative melanin content was detected at 405 nm using a micro plate reader. Kojic acid and oxyresveratrol were used as positive controls.

## 4. Conclusions

In conclusion, the results from the current study suggest that the enzyme-based assay using murine tyrosinase from B16-F10 melanoma cells is more appropriate than the mushroom tyrosinase for screening of natural compounds with potential whitening effects. The B16-F10 cells are also good for a cell-based assay for evaluating the direct effect of test compounds on melanin synthesis. Among all flavonoids from *Dalbergia parviflora* used in this test, (6a*R*,11a*R*)-3,8-dihydroxy-9-methoxy pterocarpan (**27**) and cajanin (**9**) showed clear inhibitory activities on both methods. However, some flavonoids, particularly duartin (**22**), showed induction of cellular melanin synthesis without significant effect on the enzyme activity. The mechanism involved in the observed action is still not clear.

## Figures and Tables

**Table 1:** Chemical structures of tested compounds from the heartwood of *Dalbergia parviflora.*

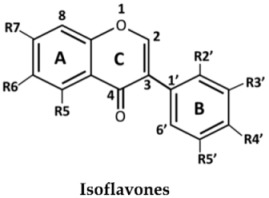	**No.**	**Isoflavones**	**R5**	**R6**	**R7**	**R2′**	**R3′**	**R4′**	**R5′**
	1	Formononetin	H	H	OH	H	H	OMe	H
	2	Calycosin	H	H	OH	H	OH	OMe	H
	3	Biochanin A	OH	H	OH	H	H	OMe	H
	4	Genistein	OH	H	OH	H	H	OH	H
	5	Khrinone B	OH	H	OH	OH	H	OMe	OH
	6	3′-*O*-Methylorobol	OH	H	OH	H	OMe	OH	H
	7	Khrinone C	OH	H	OH	OMe	OH	OMe	H
	8	Tectorigenin	OH	OMe	OH	H	H	OH	H
	9	Cajanin	OH	H	OMe	OH	H	OH	H
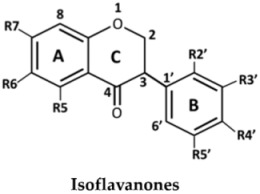	**No.**	**Isoflavanones**	**R5**	**R6**	**R7**	**R2′**	**R3′**	**R4′**	**R5′**
	10	(3*R*)-7,3′-Dihydroxy-4′-methoxy-isoflavanone	H	H	OH	H	OH	OMe	H
	11	Onogenin	H	H	OH	OMe	H	OCH_2_O	
	12	Dalparvin	H	H	OH	OMe	H	OMe	OH
	13	Dalparvin B	H	H	OH	OH	OMe	OMe	H
	14	(3*S*)-Sativanone	H	H	OH	OMe	H	OMe	H
	15	(3*R*,*S*)-3′-*O*-Methylviolanone	H	H	OH	OMe	OMe	OMe	H
	16	(3*R*,*S*)-Kenusanone G	OH	H	OH	H	OH	OMe	H
	17	(3*S*)-Secundiflorol H	OH	H	OH	OMe	OH	OMe	H
	18	Dalparvin A	OH	H	OH	OMe	H	OMe	OH
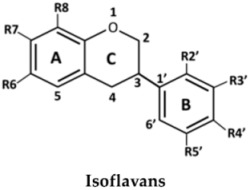	**No.**	**Isoflavans**	**R6**	**R7**	**R8**	**R2′**	**R3′**	**R4′**	**R5′**
	19	(3*R*)-Vestitol	H	OH	H	OH	H	OMe	H
	20	(3*R*)(+)-Mucronulatol	H	OH	H	OMe	OH	OMe	H
	21	(3*R*,*S*)-3′-Hydroxy-8-methoxy vestitol	H	OH	OMe	OH	OH	OMe	H
	22	Duartin	H	OH	OMe	OMe	OH	OMe	H
	23	(3*S*)-8-Demethylduartin	H	OH	OH	OMe	OH	OMe	H
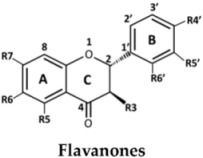	**No.**	**Flavanones**	**R3**	**R5**	**R6**	**R7**	**R4′**	**R5′**	**R6′**
	24	(2*S*)-Liquiritigenin	H	H	H	OH	OH	H	H
	25	(2*S*)-Naringenin	H	OH	H	OH	OH	H	H
	26	Alpinetin	H	OMe	H	OH	H	H	H
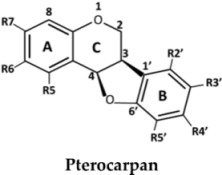	**No.**	**Pterocarpan**	**R5**	**R6**	**R7**	**R2′**	**R3′**	**R4′**	**R5′**
	27	(6a*R*,11a*R*)-3,8-Dihydroxy-9-methoxy pterocarpan	H	H	OH	H	OH	OMe	H

**Table 2:** Mushroom tyrosinase inhibitory activities of flavonoids extracted from *Dalbergia parviflora.*

No.	Compound	Mushroom Tyrosinase Inhibition	
		% Inhibition (at 200 μM)	*IC_50_* (μM)
(control)	Oxyresveratrol	98.4 ± 1.1	0.19 ± 0.1
(control)	Kojic acid	93.4 ± 1.7	16.8 ± 4.6
27	(6a*R*,11a*R*)-3,8-Dihydroxy-9-methoxy pterocarpan	84.6 ± 0.6	16.7 ± 5.0
5	Khrinone B	72.7 ± 2.2	54.0 ± 6.0
21	(3*R*S)-3′-Hydroxy-8-methoxy vestitol	64.1 ± 1.3	67.8 ± 5.8
9	Cajanin	65.0 ± 1.6	67.9 ± 6.2
10	(3*R*)-7,3′-Dihydroxy-4′-methoxy-isoflavanone	52.1 ± 0.4	176.7 ± 16.3
24	(2*S*)-Liquiritigenin	52.1 ± 1.4	178.1 ± 14.0
20	(3*R*)(+)-Mucronulatol	48.3 ± 1.6	228.9 ± 22.2
17	(3S)-Secundiflorol H	44.0 ± 3.7	278.1 ± 54.5
26	Alpinetin	36.9 ± 0.5	450.0 ± 48.5
19	(3*R*)-Vestitol	35.6 ± 2.2	473.0 ± 60.9
12	Dalparvin	31.6 ± 0.5	906.1 ± 43.6
6	3′-*O*-Methylorobol	14.3 ± 1.1	N.D.
3	Biochanin A	9.2 ± 0.4	N.D.
1	Formononetin	10% (at 300 μM)	N.D.
7	Khrinone C	2% (at 300 μM)	N.D.
2	Calycosin	0% (at 300 μM)	N.D.
11	Onogenin	0% (at 300 μM)	N.D.
15	(3*R,S*)-3′-*O*-Methylviolanone	0% (at 300 μM)	N.D.
13	Dalparvin B	4% (at 500 μM)	N.D.
14	(3*S*)-Sativanone	13% (at 500 μM)	N.D.
16	(3*R*,*S*)-Kenusanone G	0% (at 500 μM)	N.D.
18	Dalparvin A	0% (at 500 μM)	N.D.
22	Duartin	0% (at 500 μM)	N.D.
4	Genistein	S*	N.D.
8	Tectorigenin	S*	N.D.
23	(3*S*)-8-Demethylduartin	S*	N.D.
25	(2*S*)-Naringenin	S*	N.D.

N.D.: not determined; S*: These flavonoids can react with mushroom tyrosinase to form a colored product that interferes with the spectrophotometric measurement.

**Table 3:** Murine tyrosinase inhibitory activity and melanogenesis of flavonoids from *Dalbergia parviflora.*

No.	Isoflavones	Murine Tyrosinase Inhibition, *%* (at 200 μM)	15 μM		30 μM	
			% Melanin Content	% Cell Viability	% Melanin Content	% Cell Viability
1	Formononetin	-	133.8 ± 2.7	96.0 ± 3.3	155.3 ± 15.4	92.8 ± 9.4
2	Calycosin	-	142.8 ± 8.2	99.1 ± 9.2	196.6 ± 6.4	101.3 ± 1.9
3	Biochanin A	-	165.2 ± 5.9	103.4 ± 4.5	168.0 ± 5.1	102.2 ± 4.8
4	Genistein	9.9 ± 3.1	165.1 ± 1.6	128.2 ± 3.5	191.7 ± 13.9	93.8 ± 5.7
5	Khrinone B	10.1 ± 3.0	84.1 ± 5.3	95.1 ± 5.4	34.7 ± 12.3	62.3 ± 15.1
6	3′-*O*-Methylorobol	-	170.1 ± 10.5	106.6 ± 6.2	215.7 ± 15.7	101.5 ± 2.7
7	Khrinone C	-	107.6 ± 0.8	93.3 ± 2.0	154.0 ± 9.9	93.4 ± 2.0
8	Tectorigenin	10.2 ± 2.9	127.6 ± 2.8	129.7 ± 3.4	152.1 ± 4.7	119.3 ± 1.3
9	Cajanin	9.2 ± 1.5	65.8 ± 7.5	101.5 ± 6.6	61.6 ± 8.9	107.2 ± 3.3
**No.**	**Isoflavanones**					
10	(3*R*)-7,3′-Dihydroxy-4′-methoxy-isoflavanone	-	95.1 ± 3.0	94.9 ± 3.9	95.2 ± 2.7	96.5 ± 3.9
11	Onogenin	-	133.5 ± 4.6	100.0 ± 5.0	169.8 ± 15.4	92.0 ± 2.0
12	Dalparvin	-	133.8 ± 9.7	117.1 ± 8.9	199.6 ± 12.1	99.7 ± 6.7
13	Dalparvin B	9.8 ± 2.8	102.0 ± 2.7	82.3 ± 5.4	99.1 ± 2.3	74.1 ± 6.4
14	(3*S*)-Sativanone	9.5 ± 1.6	124.2 ± 4.3	106.7 ± 10.6	127.6 ± 6.0	64.3 ± 23.7
15	(3*R,S*)-3′-*O*-Methylviolanone	-	143.3 ± 8.4	74.2 ± 7.8	157.2 ± 11.5	72.9 ± 6.2
16	(3*R,S*)-Kenusanone G	-	132.6 ± 5.1	124.0 ± 1.7	158.5 ± 13.5	102.2 ± 2.8
17	(3S)-Secundiflorol H	-	117.6 ± 17.7	109.4 ± 6.6	146.4 ± 6.1	102.7 ± 4.1
18	Dalparvin A	-	132.0 ± 4.4	105.4 ± 4.8	184.2 ± 10.6	100.4 ± 7.1
**No.**	**Isoflavans**					
19	(3*R*)-Vestitol	12.4 ± 3.3	186.1 ± 1.6	142.1 ± 3.0	232.0 ± 9.3	119.7 ± 4.1
20	(3*R*)(+)-Mucronulatol	12.8 ± 2.1	178.6 ± 15.9	103.6 ± 6.9	263.7± 8.2	102.5 ± 2.7
21	(3*R*S)-3′-Hydroxy-8-methoxy Vestitol	11.4 ± 2.5	97.0 ± 6.3	102.9 ± 1.0	36.3 ± 0.1	62.4 ± 3.2
22	Duartin	-	301.1 ± 2.3	139.9 ± 7.5	326.8 ± 2.1	94.4 ± 2.8
23	(3*S*)-8-Demethylduartin	-	87.8 ± 2.0	93.5 ± 3.0	72.7 ± 4.3	98.3 ± 5.4
**No.**	**Flavanones**					
24	(2*S*)-Liquiritigenin	13.2 ± 2.7	102.9 ± 5.5	98.9 ± 10.7	124.5 ± 2.0	100.0 ± 3.5
25	(2*S*)-Naringenin	-	132.5 ± 14.5	94.3 ± 2.0	152.1 ± 3.8	98.1 ± 0.5
26	Alpinetin	-	89.7 ± 0.4	77.0 ± 12.7	92.3 ± 1.6	77.1 ± 10.2
**No.**	**Pterocarpan**					
27	(6a*R*,11a*R*)-3,8-Dihydroxy-9-methoxy pterocarpan	29.2 ± 2.9	41.9 ± 0.4	105.7 ± 4.9	53.5 ± 0.4	14.5 ± 0.9
	**Others**					
Control	Kojic acid	73.8 ± 1.0	68.9 ± 5.2	98.9 ± 4.7	74.2 ± 5.8	99.6 ± 2.3
Control	Oxyresveratrol	99.4 ± 1.0	24.3 ± 1.1	117.2 ± 2.2	28.4 ± 1.7	118.1 ± 5.1

-: no inhibition at 200 μM.
